# The effect of different osseodensification techniques on implant stability in the maxillary anterior esthetic zone. A split-mouth randomized clinical trial

**DOI:** 10.1186/s12903-026-08802-9

**Published:** 2026-06-05

**Authors:** Marwan Adel Ali, Ahmed Mostafa ElSabbagh, Yehia El-Mahallawy

**Affiliations:** Oral and Maxillofacial Surgery Department, Faculty of Dentistry, Alexandria University, Alexandria, Egypt

**Keywords:** Dental implant, Endosseous, Implant stability, Osseointegration, Osseodensification, Densah bur, Magnetic mallet, Linear models

## Abstract

**Objectives:**

To clinically and radiographically compare two osseodensification techniques in terms of implant stability and osseointegration in a split-mouth design.

**Materials and methods:**

7 patients with an edentulous maxillary anterior area who need implant placement were divided into 2 groups. Group A included 7 implants and was prepared using the Densah Bur technique (DB-Group), while Group B included 7 implants and was prepared using the Magnetic Mallet technique (MM-Group). Surgery was performed, followed by clinical and radiographic follow-up to evaluate implant stability in both groups. A linear mixed model (LMM) statistical analysis was utilized to handle the clustering effect of the data.

**Result:**

The mixed model revealed a stable data analysis owing to the significant intercept for all of the dependent variables (*P* < 0.001). Densah Burs technique demonstrated lower postoperative pain, faster wound healing, higher implant stability, and greater preservation of labial bone thickness, while both methods showed similar long-term bone density and crestal width outcomes.

**Conclusion:**

Within the confines of this study, the rotary-based Densah Bur osseodensification technique was associated with higher implant stability and labial plate thickness, alongside comparable bone density. These findings suggest potentially more predictable osteotomy compaction and autografting in the maxillary anterior esthetic zone implant osteotomy preparation.

**Trial registration:**

Trial was retrospectively registered at pactr.samrc.ac.za [PACTR202412717492416-(2024-12-31)]

**Supplementary Information:**

The online version contains supplementary material available at 10.1186/s12903-026-08802-9.

## Introduction

The basic principle in dental implant placement is the capability of the implant bed preparation to preserve the biological integrity of the alveolar bone at the structural and cellular level in order to attain a direct living bone-to-functional metal interface. Ultimately, this biological stability provides the essential scaffold for a predictable esthetic outcome [1, 2]. Osseodensification (OD) is a contemporary concept for implant bed osteotomy preparation, which has been at the forefront of the revolution in surgical site preparation in implantology [2, 3].

The prevalent OD technique involves a special drill in a non-cutting and low-plastic deformation process, allowing autografting of the osteotomy walls and implant bed preparation in a non-excavating manner [4]. Another OD modality involves the utilization of magneto-dynamic technology in implant-bed preparation [5]. This magneto-dynamic technology is based on the electromagnetism physical principle, where controlled forces are applied to the body with a steady time of impact. This technology is utilized in a magnetic mallet (MM) device, which produces a shock wave in a time-controlled manner, exerting a controlled force while minimizing the time of impact [5, 6]. The magneto-dynamic technology has several applications in the dental field, with different shock-wave utilization, stating force modes of 75, 90, 130, and 260 daN and 80µs time of impact [5, 6].

The study aims to evaluate and compare the changes in implant stability using two different OD approaches: the rotary Densah bur and the Magnetic Mallet techniques. The specific aim of this trial was to design and implement a blinded split-mouth study in the maxillary anterior esthetic zone. It was hypothesized that Densah bur and the Magnetic Mallet result in comparable outcomes regarding implant stability in the maxillary anterior esthetic zone. The secondary aim of the study was to evaluate the osseointegration radiographically between two techniques.

## Materials and methods

### Study design

**Fig. 1:**
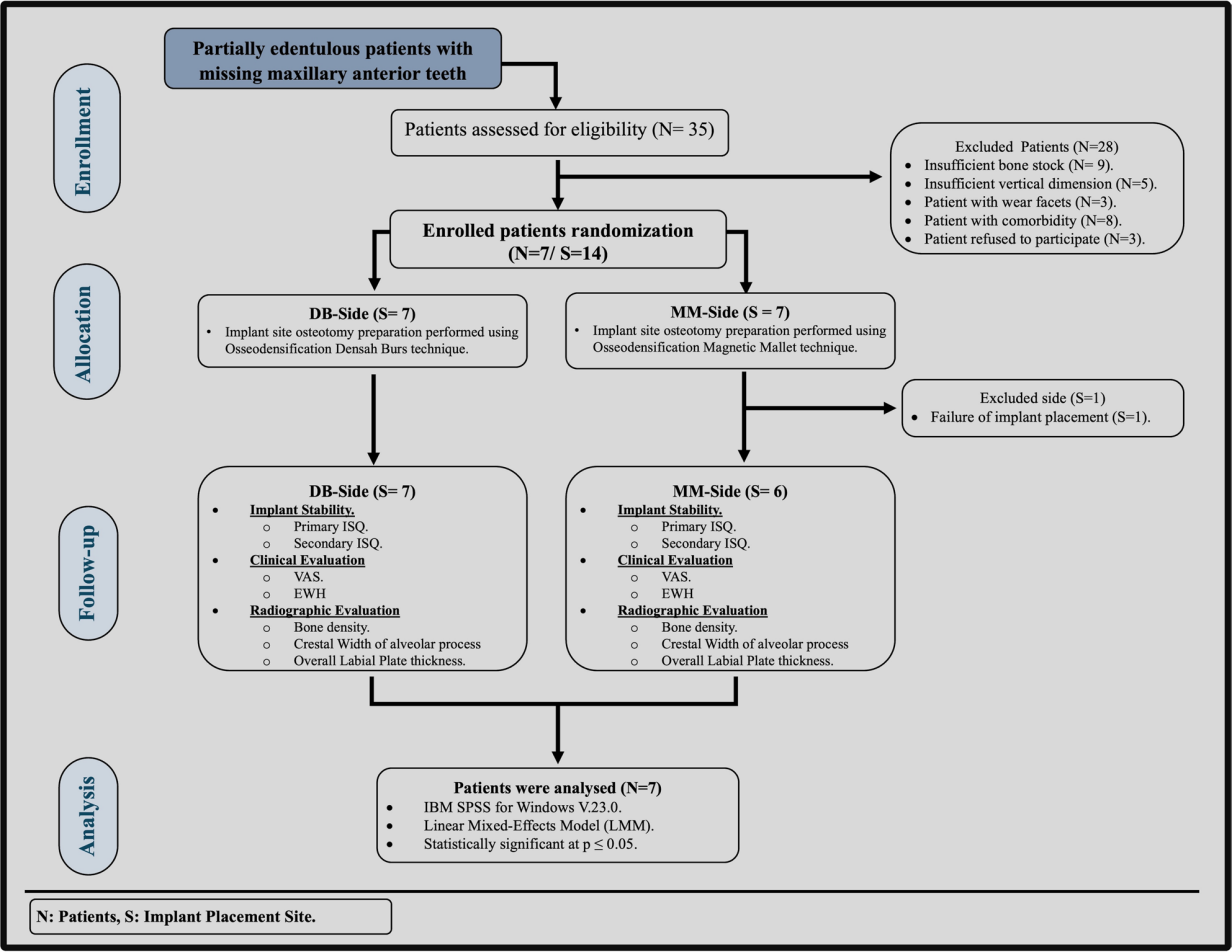
CONSORT flow chart

This study was a split-mouth randomized clinical trial comparing different osseodensification approaches for implant placement in the maxillary anterior esthetic zone. This trial was organized and reported according to the CONSORT guidelines (http://www.consort-statement.org) (Fig. 1). The clinical report was conducted under the doctrines of the Helsinki guidelines after approval from the local Research Ethical Committee (IRB:0902-4/2024/IORG:0008839) (Pactr.Samrc.ac.za/PACTR202412717492416/ 31-12-2024). Sample size was estimated based on assuming a 95% confidence level and 80% study power. Cohort size estimation was calculated based on the Rosner method, using the G*Power 3.1.9.7 software [7, 8]. The mean primary implant stability was reported to be 71.77 ± 2.71 for the Magnetic Mallet group [6] and 65.17 ± 4.39 for the Densah bur group [9]. Based on the difference between the two dependent means using the highest SD 4.39, to ensure enough study power, a sample of 6 implants was required, yielding an effect size of 1.503. This was increased to 7 implants to compensate for the lost follow-up cases. Total sample size = number per side × number of sides = 7 × 2 = 14 sides in 7 patients.

### Patients’ selection

The study included adult partially edentulous patients in the maxillary anterior esthetic zone with missing maxillary anterior teeth that needed multiple dental implants for dental rehabilitation, Prosthodontic Diagnostic Index (PDI) Class I [10]. The minimum available alveolar bone stock required for implant placement was set at 10 mm and 5 mm for bone length and width, respectively [11]. Individuals suffering from any systemic morbidities, bone diseases, or parafunctional habits were excluded from the study. Participants were drafted from the Outpatient Clinic of the Oral and Maxillofacial Surgery Department, Faculty of Dentistry, Alexandria University, from April 2024 to April 2025.

Patients were informed about the procedure, its benefits, and challenges. Before embarking on the study, they presented a written informed consent for both study participation and clinical and radiographic records utilization after proper deidentification. For each enrolled patient, side randomization was performed using a permuted block size of 2, generated using a computer-generated randomization software (http://www.randomizer.org/*)*, and with a 1:1 allocation ratio. Allocation concealment was performed using the Sequentially Numbered Opaque Sealed Envelopes (SNOSE) technique. Implant placement sites were given a serial number that would be used in the group allocation and the starting implant placement technique. These numbers were written on identical sheets of paper with the group to which each implant site was allocated. The paper was placed inside opaque envelopes. Correspondingly, the other implant site was then allocated to the alternate technique. A trial independent personnel (H.A) was assigned the role of keeping the envelopes and unfolding them only at the time of the operation so that side allocation and starting implant placement technique were concealed from the operator. The available implant placement sites in the included patients were randomly divided into 2 equal groups; implant site osteotomy preparation performed using Osseodensification Densah Burs technique was included in the *DB-Group*, while those prepared using Magnetic Mallet Osseodensification were included in the *MM-Group.*

### Preoperative assessment & surgical phase

A comprehensive preoperative assessment was conducted through proper history taking, thorough clinical and radiographic examination. A preoperative Cone Beam Computed Tomography (CBCT) scan was obtained for each patient (Carestream Cs9600 Scanner, Carestream Dental LLC, Atlanta, GA.). Implant placement sites were methodologically scrutinized on the topographic scan by two experienced implant practitioners (A.E. / Y.E.). The Preoperative alveolar bone stock was reviewed, inspecting the available bone length, width, arch vertical & mesio-distal dimensions, and bone quality, according to Misch’s radiographic classification [12]. Preoperative scaling and root planning were performed, and oral hygiene recommendations and instructions were given to all of the enrolled patients.

The surgical procedure was preceded by prophylactic Amoxicillin (875 mg) + clavulanate (125 mg) antibiotic half an hour before surgery (Augmentin, GlaxoSmithKline, UK), and hot antiseptic chlorhexidine HCl 125 mg/100 ml mouthwash before surgery to eradicate any oral contamination ( Hexitol, Arab drug company, Cairo, Egypt). All procedures were performed in a dental chair after Local scrubbing of the implant placement sites with povidone-iodine scrub solution, and with 4% Articaine + 1:100,000 epinephrine infiltration anesthesia (Alexadricaine, Alexandria, Egypt). The surgical procedure was conducted by a single operator for all the cases and for both sides, with proficient experience in the utilization of both osseodensification techniques (M.A). A full-thickness mucoperiosteal crestal flap was raised and reflected for the exposure of the implant placement sites. Subsequently, the concealment envelope was opened to allow implant site group allocation (H.A).

For Implant sites in the DB-Group [6], the initial osteotomy was conducted using the Densah-Pilot Drill-G3 (Apical ⌀: 1.6 mm) in a forward Clockwise cutting direction at 800-RPM and 30 N/cm-torque under copious irrigation. To standardize the procedure, all subsequent Densah drills were operated in a reverse counter-clockwise densifying mode at 800-RPM and copious irrigation once the preplanned osteotomy depth was reached. Osteotomy sites were progressively enlarged and densified, commencing with the Densah Bur-VT1525 (Apical ⌀: 2 mm). Implant site osseodensification was continued until the planned implant site diameter was obtained. The selection of the final drill was based on the final regular drill size of the selected implant. Conclusively, implants were then inserted in a 1 mm sub-crestal position (IS-III-CMI Implant, Neobiotech, Seoul, Korea).

For Implant sites in the MM-Group [6], the initial site preparation was conducted using the first pilot osteotome (Bore ⌀: 1.8 mm) on Mode 1 (~ 75 daN). This mode delivers brisk, controlled tap with the force of a 75 kg weight, but concentrated into an 80-microsecond pulse. Subsequently, the osteotomy sites were progressively enlarged, beginning with the 100P-Osteotome (Apical ⌀: 1.0 mm, Coronal ⌀: 3.1 mm ) at Mode 2 (~ 90 daN), which delivers each magnetic mallet impulse as a rapid, controlled strike equivalent to the force exerted by a 90 kg mass, concentrated into an 80 microsecond pulse. These parameters were selected in accordance with the manufacturer’s recommendation for D2-D3 bone densities in the maxillary anterior zone. Selecting the final drill was based on the final regular drill size of the selected implant. Implant site osseodensification was continued until the planned implant site diameter was obtained. Implants were then inserted in osteotomy sites in a 1 mm sub-crestal position (IS-III-CMI Implant, Neobiotech, Seoul, Korea).

For Both Implant placement sites, assessment and recording of primary implant stability using the Radio Frequency Analysis (RFA) with its incorporated SmartPeg (3rd gen. Osstell™ ISQ). Primary implant stability ISQ values were obtained by a blinded operator (Y.E.). This was followed by securing the cover screw for each implant and closure of the incised wound using interrupted 3/0-black silk suture material. Patients were instructed to continue the preoperatively administered antibiotic for 5 postoperative days, along with the prescription of a Diclofenac potassium t.i.d for 3 postoperative days (50 mg, Cataflam, Novartis Switzerland).

### Implant stability assessment and prosthetic phase

Secondary implant stability analysis was performed 6 months after implant placement. RFA was performed using the Osstell™ ISQ device (Integration Diagnostic Ltd. Company, Sävedalen, Sweden) [13]. Infiltration local anesthesia was administered, and small incisions were made for the retrieval of the cover screw and securing the SmartPeg for implant stability analysis. The final prosthesis was delivered 6 months after implant placement for all of the enrolled patients. Once again, Primary and secondary implant stability ISQ values were obtained by a blinded operator (Y.E.).

### Clinical follow-up criteria

Clinical follow-up was performed after 7, 14, and 21 days. Subjective Assessment of postoperative pain scores was evaluated and compared between both sides using a 10-point Visual Analogue Scale (VAS). The patients were asked about the pain at each implant site, and not the overall pain of the procedure [9]. Monitoring patient oral hygiene was done to follow up on patient compliance with oral hygiene instructions [9]. Wound healing was quantitatively monitored at each implant site using the Early Wound Healing (EWH) score. The EWH-score is an objective quantitative wound healing analysis scale [14]. EWH-score provided the objective analysis of 3 clinical parameters: Clinical signs of Re-epithelization (CSR), Clinical signs of Hemostasis (CSH), and Clinical signs of Inflammation (CSI). The value of each parameter was obtained, and the summation generates the EWH-score for each implant site at each follow-up session. The EWH-score values range from 0 to 10, where a 10 EWH-score represents ideal wound healing, while a 0 EWH-score represents the worst wound (Supplementary Fig. 1).

### Radiographic outcome criteria

Radiographic appraisal was performed by obtaining immediate and 6-month postoperative CBCT scans. The immediately postoperative scan was obtained during the first postoperative week. To ensure longitudinal accuracy, both scans were obtained using a standardized phantom calibrated machine, which was also used for the preoperative scan. Radiographic assessment was conducted by a blinded operator (A.S.) Peri-implant bone density was measured using a Region Of Interest (ROI) tool of the On-Demand 3D software (On-Demand 3D APP-DBM, Cybermed, Seoul, South Korea). A standardized 1*1 ROI was utilized across multiple slices at three specific locations relative to the implant interface. These specific locations were 2 mm apical to the implant platform from the buccal and lingual aspects, and 2 mm below the implant apex from the apical aspect. This standardization positioning ensured consistency across both scans. Measurements were collected, and the mean value was obtained. The value obtained in the immediate postoperative scan was compared to that obtained in the 6-month scan [9]. Values were obtained in the standard CBCT Grey Scale Value (GSV). For data reproducibility, the GSV to Hounsfield units (Hu) conversion formula was utilized. The formula to equivalent bone density was determined as: Y = 0.682 (x) – 161, where Y = bone density and X=CBCT gray value [15].

The width of the alveolar bone at its crestal edge surrounding the implant was measured in the immediate and the 6-month postoperative scans [9]. The Bucco-Palatal ridge dimensions were measured using the ruler measurement tool on the On-Demand 3D software. Assessment of the thickness of the labial plate was performed in the preoperative and the 6-month scans to evaluate the overall survival of the gained labial plate thickness following the osseodensification procedure [16].

### Statistical analysis

Statistical data analysis was conducted using the IBM-SPSS v.23 for Windows OS (Armonk, NY: IBM Corp). The significance of the obtained results was judged at the 5% level. Data normality was checked using the Shapiro-Wilk test and Q-Q plots. The split mouth design has an inherent clustering effect as several implants are placed in the same patient. To address this inherent nesting, a 2-level hierarchical structure was set in the study, in which the individual implant site (*N* = 14) represented the lower level of analysis (Level 1), and the patient (*N* = 7) represented the upper-level clustering variable (Level 2), with each patient contributing one test and one control site. Accordingly, a Linear Mixed-Effects Model (LMM) was utilized using Restricted Maximum Likelihood (REML) estimation to evaluate the intra-group correlation and to handle the unbalanced data structure [17]. The LMM model included the groups (intervention) and the time (follow-up interval) as fixed effects, and a random intercept for the subject (Patient ID) to accommodate the clustering of two sites within the same individual, as well as repeated measures. Model selection was guided by the Akaike Information Criterion (AIC) and Bayesian Information Criterion (BIC), which confirmed the Random Intercept structure as the most stable fit. The model used the F-test with the Kenward-Roger approximation for estimating degrees of freedom and more accurate p-values. Pairwise comparisons between levels of time and intervention were carried out directly within the LMM using Bonferroni correction to adjust for multiple testing and reduce the risk of Type I error. To ensure statistical stability against data imbalance, a paired sensitivity analysi

s of the primary outcome was implemented alongside the baseline model to confirm the robustness of the fixed effects.

## Results

A total of seven patients were included in this study. The mean age of the seven patients was 37.71 ± 6.65 years. In terms of gender distribution, a 6:1 male-to-female ratio was reported. A comprehensive tabulation of the cases is presented in Supplementary Table 1. In one of the cases, a failure for implant placement was encountered in the MM-group, while normal implant placement was attained in the DB-side. In this failed implant, mallet application was conducted in a normal manner; however, during implant insertion, a crack propagated to the labial plate, causing its dislodgment and fracture. To handle this data imbalance caused by the single implant failure, the LMM was utilized using REML estimation. For all of the investigated dependent variables, a highly significant effect was observed for the LMM intercept (*P* < 0.001), reflecting model stability. Furthermore, a sensitivity analysis for the primary outcome (Implant stability) to evaluate the statistical impact of this failed case was conducted. The sensitivity analysis confirms that statistical choice is methodologically robust and not skewed by the handling of the failed implant. The outcome of the sensitivity analysis is presented in the supplementary data.

### Clinical dependent variables

**Fig. 2:**
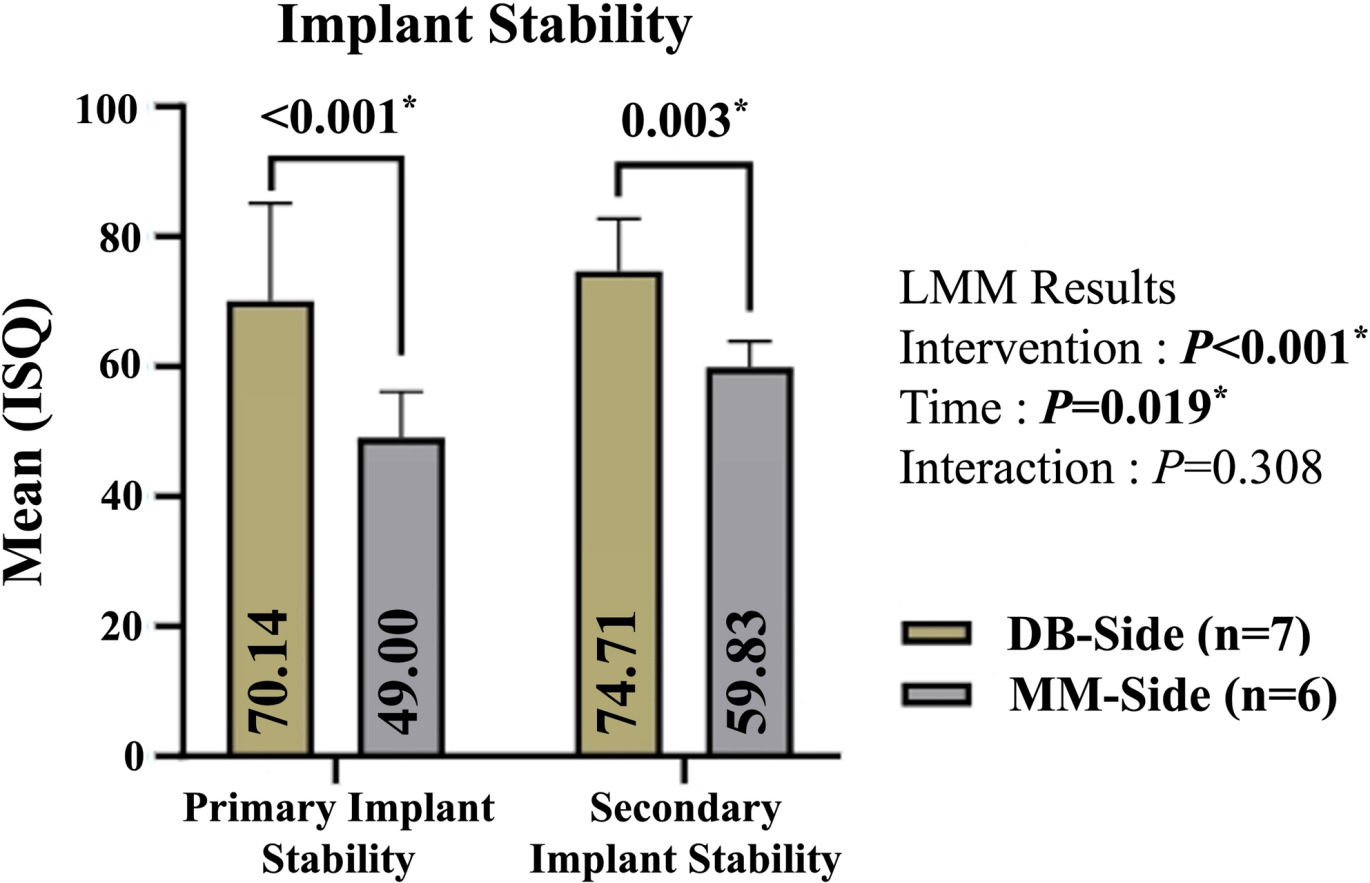
Effect bar representing the Implant Stability (ISQ) across the follow-up period in the two Osseodensification groups

The mean ± SD primary and secondary ISQ values are reported in Table 1. The LMM for the ISQ as dependent variable revealed that a statistically significant effect for both the intervention group (*P* < 0.001^*^) and time (*P* = 0.019^*^); however, the group-time interaction was statistically insignificant (*P* = 0.308) (Table 2) (Fig. 2). On both sides, a predictable and significant reduction in the mean VAS-score over the clinical follow-up period was reported, with lower mean scores in the DB-side (Supplementary Table 2).

**Table 1:** Implant stability (ISQ) values in the two Osseodensification groups

ISQ		DB (*n* = 7)	MM (*n* = 6)	*P* ^1^
Primary Implant Stability	Mean ± SD	70.14 ± 14.99	49.00 ± 7.13	< 0.001*
Secondary Implant Stability		74.71 ± 8.10	59.83 ± 4.07	0.003*
***P*** ^**2**^		0.274	0.024*	

*Statistically significant difference at *p* value < 0.05

**Table 2:** Fixed effects of the two interventions, time, & their interaction on Implant Stability

Parameters	N-df	D-df	F test	*P*
Intercept	1	6.29	544.52	< 0.001*
Groups (Interventions).	1	17.20	4.13	< 0.001*
Time.	2	16.39	6.71	< 0.019*
Groups x Time (Interaction).	2	16.39	1.11	0.308

*Statistically significant difference at *P* < 0.05

*N-df*: Numerator degree of freedom, *D-df*: Denominator degree of freedom

**Fig. 3:**
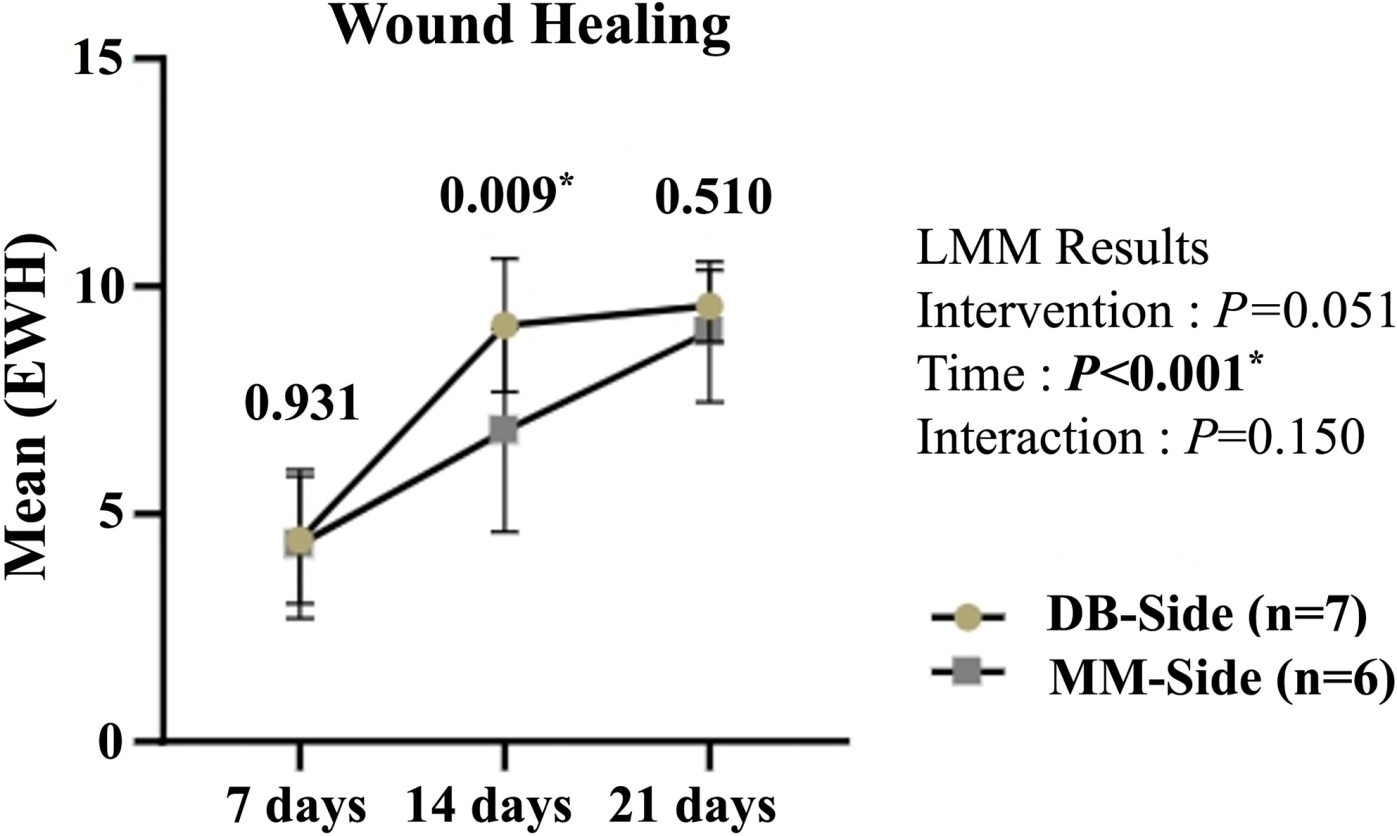
Effect plot representing the wound healing (EWH) across the follow-up period in the two Osseodensification groups

Regarding the postoperative wound healing, the mean ± SD EWH-score outcome across the follow-up period is reported in Table 3. The LMM for the EWH-score as dependent variable revealed that a borderline significant effect for the intervention group (*P* = 0.05^*^), which significantly changed across time (*P* < 0.001^*^); however, the group-time interaction was statistically insignificant (*P* = 0.150) (Table 4; Fig. 3).

**Table 3:** Wound Healing (EWH) values in the two Osseodensification groups

EWH		DB (*n* = 7)	MM (*n* = 6)	*P* ^1^
**7th day**	Mean ± SD	4.43 ± 1.40	4.33 ± 1.63	0.931
**14th day**		9.14 ± 1.46	6.83 ± 2.23	0.009*
**21st day**		9.57 ± 0.79	9.00 ± 1.55	0.510
***P*** ^***2***^		< 0.001*	< 0.001*	

*Statistically significant difference at *p *value < 0.05

**Table 4:** Fixed effects of the two interventions, time, & their interaction on wound healing scores

Parameters	N-df	D-df	F test	*P*
Intercept	1	6.07	598.70	< 0.001*
Groups (Interventions).	1	29.27	4.13	0.051
Time.	2	27.40	38.63	< 0.001*
Groups x Time (Interaction).	2	27.40	2.03	0.150

*Statistically significant difference at *P* < 0.05

*N-df*: Numerator degree of freedom, *D-df*: Denominator degree of freedom

### Radiographic dependent variables

**Fig. 4:**
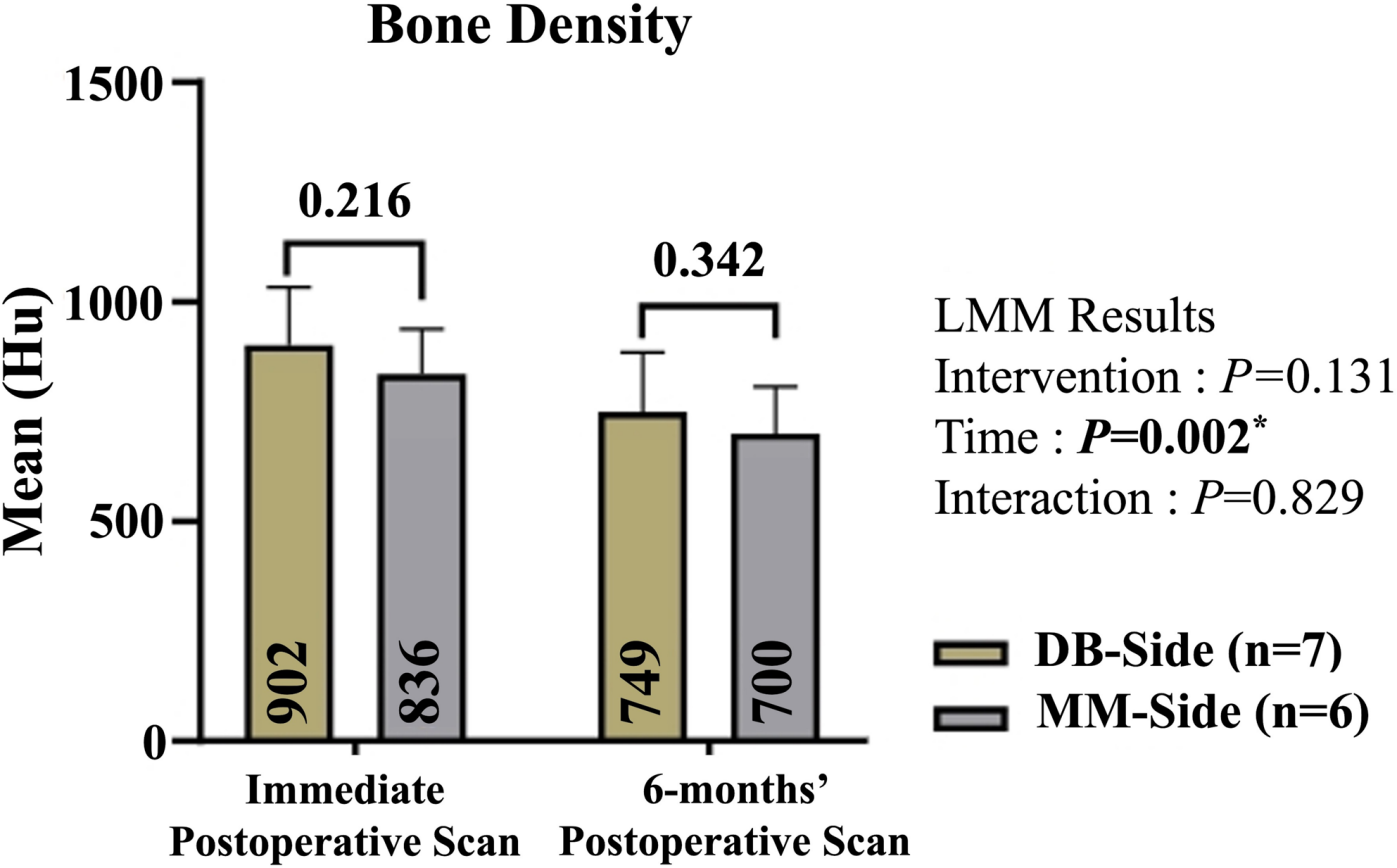
Effect bar representing the peri-implant bone density (Hu) across the follow-up period in the two Osseodensification groups

The mean ± SD bone density outcome across the follow-up period is reported in Table 5. Inter-group difference in both the immediate and 6-month scans was statistically non-significant (*P* = 0.216 / *P* = 0.342). Intra-group comparisons revealed a statistically significant reduction in bone density over time within both groups (P = *0.012* for the DB-side, and *P* = 0.033 for the MM-side). The LMM for the peri-implant bone density as dependent variable revealed that a statistically significant effect for the time (*P* < 0.002^*^); however, the effect of the intervention group and the group-time interaction were statistically insignificant (P = *0.131*) (*P* = 0.829) (Table 6; Fig. 4).

**Table 5:** Peri-implant Bone Density (Hu) values in the two Osseodensification groups

Hu		DB (*n* = 7)	MM (*n* = 6)	*P* ^1^
Immediate Postoperative	Mean ± SD	901.86 ± 131.65	835.67 ± 102.14	0.216
6-months’ Postoperative		749.00 ± 134.76	700.17 ± 106.19	0.342
***P*** ^**2**^		0.012*	0.033*	

*Statistically significant difference at *p* value < 0.05

**Table 6:** Fixed effects of the two interventions, time, & their interaction on bone density

LMM-Parameters	N-df	D-df	F test	*P*
Intercept	1	5.96	605.27	< 0.001*
Groups (Interventions).	1	17.08	2.52	0.131
Time.	1	16.12	13.22	0.002*
Groups x Time (Interaction).	1	16.12	0.05	0.829

*Statistically significant difference at *P* < 0.05

*N-df*: Numerator degree of freedom, *D-df*: Denominator degree of freedom

**Fig. 5:**
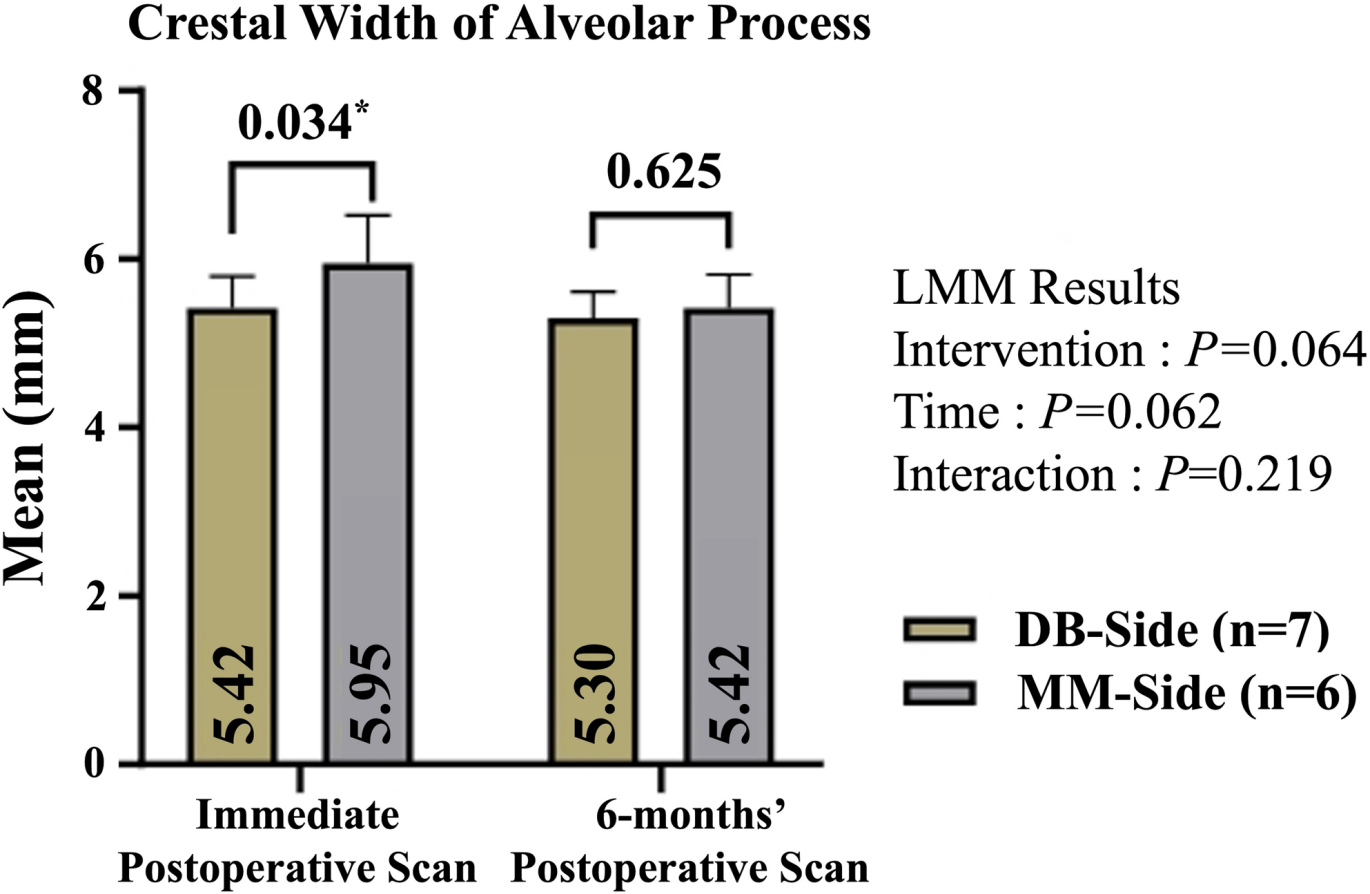
Effect bar representing the crestal width of the alveolar process (mm) across the follow-up period in the two Osseodensification groups

The mean Labio-palatal crestal width of the alveolar process measurement across the follow-up period is reported in Table 7. The LMM for the crestal width of the alveolar bone as dependent variable revealed that the effect of the intervention group (*P* = 0.064), time (*P* = 0.062), and their interaction (*P* = 0.219) were statistically insignificant (*P* = 0.197) (*P* = 0.937) (Table 8; Fig. 5).

**Table 7:** Crestal width of the alveolar process (mm) values in the two Osseodensification groups

mm		DB (*n* = 7)	MM (*n* = 6)	*P* ^1^
Immediate Postoperative	Mean ± SD	5.42 ± 0.38	5.95 ± 0.57	0.034*
6-months’ Postoperative		5.30 ± 0.31	5.42 ± 0.40	0.625
***P*** ^**2**^		0.603	0.040*	

*Statistically significant difference at *p* value < 0.05

**Table 8:** Fixed effects of the two interventions, time, & their interaction on crestal width of the alveolar process

LMM-Parameters	N-df	D-df	F test	*P*
Intercept	1	5.88	4176.72	< 0.001*
Groups (Interventions).	1	17.58	3.92	0.064
Time.	1	16.29	4.00	0.062
Groups x Time (Interaction).	1	16.29	1.64	0.219

*Statistically significant difference at *P* < 0.05

*N-df*: Numerator degree of freedom, *D-df*: Denominator degree of freedom

**Fig. 6:**
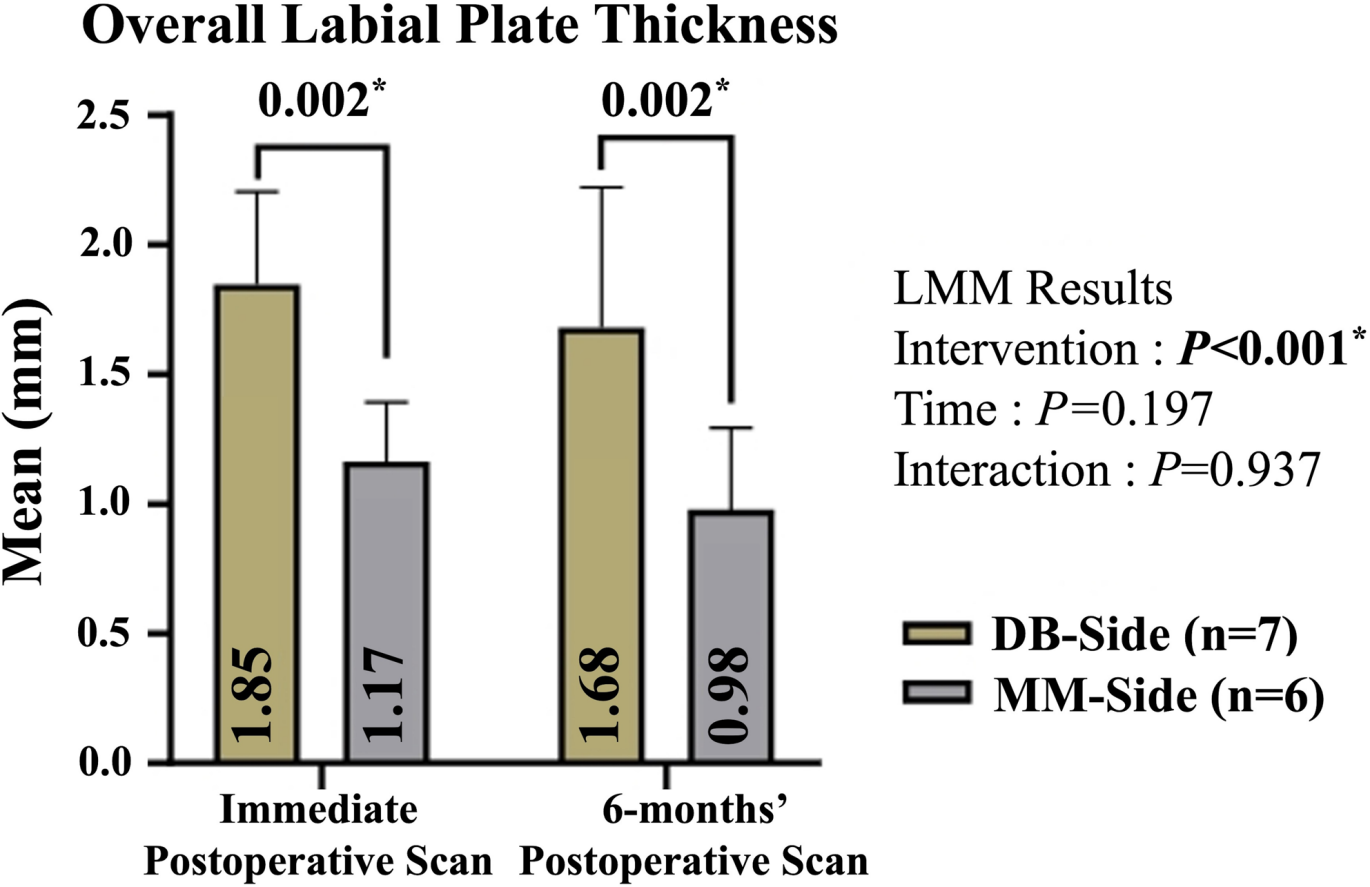
Effect bar representing the overall labial plate thickness (mm) across the follow-up period in the two Osseodensification groups

The overall mean ± SD labial plate thickness outcome across the follow-up period is reported in Table 9. The LMM for the overall labial plate thickness as dependent variable revealed that a statistically significant effect for the intervention group (*P* < 0.001^*^); however, the effect of the time and the group-time interaction were statistically insignificant (*P* = 0.197) (*P* = 0.937) (Table 10; Fig. 6).

**Table 9:** Overall labial bone thickness (mm) values in the two Osseodensification groups

mm		DB (*n* = 7)	MM (*n* = 6)	*P* ^1^
Immediate Postoperative	Mean ± SD	1.85 ± 0.36	1.17 ± 0.23	0.002*
6-months’ Postoperative		1.68 ± 0.54	0.98 ± 0.32	0.002*
***P*** ^**2**^		0.365	0.345	

*Statistically significant difference at* p* value < 0.05

**Table 10:** Fixed effects of the two interventions, time, and their interaction on overall labial plate thickness

LMM-Parameters	N-df	D-df	F test	*P*
Intercept	1	6.26	211.26	< 0.001*
Groups (Interventions).	1	17.47	26.33	< 0.001*
Time.	1	16.46	1.81	0.197
Groups x Time (Interaction).	1	16.46	0.01	0.937

*Statistically significant difference at *P* < 0.05

*N-df*: Numerator degree of freedom, *D-df*: Denominator degree of freedom

## Discussion

Implant placement in the maxillary anterior esthetic zone is constrained by limited available bone stock, thin labial plate, and high esthetic demands [17]. Achieving adequate primary stability and successful osseointegration while preserving labial bone and soft tissue architecture are an essential factors for long-term implant success [17]. Osseodensification techniques offer bone-preserving osteotomy preparation, with compaction rather than excavation, making it a favorable choice in the esthetic zone [5, 6]. This split-mouth, randomized trial compares different osseodensification approaches for maxillary anterior implant placement.

The split-mouth nature of the data opted for an LMM analysis to handle the imbalance and to prevent the clustering effect in the regular analysis [18]. For all of the investigated dependent variables, a highly significant intercept effect was reported (*P* < 0.001), reflecting model stability. The LMM intercept effect is the baseline for the model, indicating the anticipated outcome of the dependent variable in the absence of any effect from the independent variables.

Two notable complications were reported in the MM-group: one labial plate fracture during osteotomy preparation that averted implant placement, and one palatal crack that occurred during preparation. No similar events were observed in the DB group. While the MM delivers a controlled longitudinal impulse, the resulting lateral expansion force is prompt and substantial. In cases of extreme ridge narrowness, the impact-driven OD may exceed the tensile strength of the cortical bone before adaptive formation can occur. Bennardo et al. reported a very low complication rate with a high implant survival rate in their systematic review regarding the utilization of MM in implant placement [5]. In this study, a steep learning curve was observed with the utilization of the magneto-dynamic implantation technique, in contrast to a shallow one in the Densah bur technique. Our experience involved a single operator with experience in implant placement.

The Densah bur technique involves regular motor handling, which is a common practice for the practitioner. The mallet involves a slow, steady pulsed application, which requires meticulous skills for handling the device. In the study, both complications occurred in the early cases, which may point to the surgeon getting accustomed to the mallet handling and application. From our experience, a flat bony platform for implant placement is easier for the MM application, which is rarely naturally attained in the anterior maxillary alveolus.

The outcome of the LMM regarding implant stability could outline that different OD techniques had a significant effect on their implant stability outcome (Group *P* < 0.001^*^), which significantly changed across the healing period (Time *P* = 0.019); however, the changes in the reported implant stability over time were similar in both groups (G*T *P* = 0.308). This could outline the ability of both modalities in achieving high ISQ values owing to the osteotomy densification processes and the increased bone-to-implant contact (BIC). Lahens et al. demonstrated, in an animal model, that bur-driven osseodensification produces significantly greater BIC and insertion torque compared to conventional drilling [19]. The clinical relevance of the rotary OD is more prominent in this report owing to bypassing the 70 ISQ threshold in both the primary and secondary stability.

The comparable outcome in both studies and the statistically significant gain in ISQ values in the MM-side, despite the reported complications, indicates that proper bone healing and remodeling were achieved in the MM-group, which has compensated for reduced primary stability. This could be attributed to the generated high-energy impact with almost no increase in the intra-osteotomy temperature, which contributes to the success of the MM in achieving a predictable physiological densification. These findings were similar to those observed by Negidah et al., who reported increasing ISQ values for MM over time [6].

The alveolus density analysis outcome indicates a significant decrease in bone density from the immediate postoperative period to the six-month follow-up within each group, which is consistent with the natural remodeling process from woven to lamellar bone, as demonstrated by Trisi et al. [20]. The bone density LMM analysis could outline that different OD techniques didn’t have a significant difference in overall bone density outcomes (Group *P* = 0.131), and that a significant reduction in bone density over time, irrespective of the intervention type, is reported (Time *P* < 0.002^*^). Additionally, the pattern of bone density change over time did not differ between the two OD techniques (G*T *P* = 0.829).

The favorable reported implant stability could be correlated with the maintenance and increase in the gauged alveolar bone density in both OD techniques. Qualitative histomorphometrical analysis for the effect of OD osteotomy preparation on bone healing in the sheep model reported bone remodeling and growth with the presence of compacted bone chips observed along the length of the osteotomy wall [21]. The primary stability achieved in both groups stems from distinct biomechanical actions. The bur-based OD utilizes a rolling-sliding contact to induce plastic deformation and autografting into trabecular spaces. In contrast, the MM relies on a fracture-and-impact compaction model with the utilization of high-energy impulses that cause micro-fractures. Both Osseodensification aids in the activation of the mechanotransduction pathway, where the mechanical strain applied during osteotomy preparation triggers a biological response within the osteocyte network. This strain-sensing stimulus releases signalling molecules, which recruit osteoblasts to the site and accelerate the transition from primary mechanical stability to secondary bone-implant biological integration [22].

Regarding the clinical performance of both OD techniques, the LMM outcomes could be outlined that the reduction in the postoperative pain was significant across the clinical follow-up regardless of the intervention type (Time *P* < 0.001^*^), with a significant difference between the osseodensification groups and the DB-side experiencing overall lower pain than the MM-side (Group *P* = 0.004^*^). Arafat and Elbaz reported a similar lower discomfort and swelling following bur-driven OD when compared to impacted-based OD [9]. However, the LMM outcome declared that both OD interventions followed a similar pattern of pain reduction across the clinical follow-up intervals (G*T *P* = 0.509). Pain-reported outcome evaluation in a split-mouth model requires acknowledging that pain is a diffuse, centrally processed phenomenon. This inherent subjectivity can make accurately differentiating discomfort between two stimulants at surgical sites difficult, potentially introducing a carry-over effect in site-specific VAS reporting.

Wound healing scores were assessed using the Early Wound Healing score (EWS). It progressed significantly in both groups, but in the DB-group, the healing scores were higher on day 14. The LMM outcome outlines that that different OD techniques had a borderline significant effect in their wound healing outcome based on the quantitative EWH-scores (Group *P* = 0.051), which significantly changed across the clinical follow-up regardless of the intervention type (Time *P* < 0.001^*^), however the pattern of wound healing outcome over time was similar, did not significantly differ, in the two intervention groups (G*T *P* = 0.150). Similar findings were observed by Marini et al., who pointed out that minimizing trauma to the surrounding tissues positively affects early wound healing scores [14]. The accelerated healing in the DB group could be attributed to reduced periosteal disruption and micro-fracturing gained with Densah Burs preparation, resulting in faster soft tissue healing and maturation.

Regarding the crestal width of the alveolar process, the LMM outcome could indicate that although the *MM-group* initially exhibited a wider ridge crest, both techniques resulted in comparable crestal bone width preservation after six months. While the MM group demonstrated a significant reduction in crestal width after 6 months, the DB-group showed decreased but maintained relative stability. These findings indicate that the expansion achieved with the mallet technique may not be sustained in the long term, while osseodensification with Densah burs appears more conservative in preserving ridge dimensions. Similar observations were reported by Arafat and Elbaz, who found that osseodensification preserved alveolar width following sinus floor elevation, whereas expansion-based techniques were associated with crestal resorption [9]. Likewise, Ghoneim et al. demonstrated significantly better preservation of crestal bone levels at six months in the Densah group compared to the Magnetic Mallet group in the esthetic zone [11].

Koutouzis et al. evaluated the bone expansion effect of OD at different levels of initial alveolar ridge width [23]. Their outcome reported that OD permits a significant and greater ridge crestal expansion in narrow ridges with adequate trabecular bone volume maintenance [23].

The minimum required crestal width for study enrollment was 5 mm, which allowed the placement of a regular platform implant. Assessment of the preservation of the labial plate thickness reported the meticulous nature of the implant placement technique and the compaction capability of the utilized OD techniques [16]. The overall LBT was significantly higher in the DB-group both immediately and six months postoperatively, demonstrating that the Densah Bur technique maintained more labial bone thickness, which is essential for anterior maxillary esthetics and long-term implant success. The LMM outcome revealed that different OD techniques have a significant difference in overall labial plate thickness (Group *P* < 0.001^*^), and that labial thickness did not significantly change over the follow-up period, irrespective of the intervention type (Time *P* < 0.197). Additionally, the pattern of change in labial thickness over time was similar between the two osseodensification techniques (G*T *P* = 0.937). Densah burs superior performance may be related to its controlled, non-percussive expansion that minimizes labial bone disruption.

The study’s split-mouth, randomized design curbed inter-subject confounders through within-patient comparisons [24]. Restricting the assessment sites to the maxillary esthetic zone minimized intra-patient variability in bone quality and ensured biomechanical symmetry. Distinct non-overlapping osteotomy locations and concealed allocation prevented carry-over and sequence biases. Additionally, LMM statistical analysis resolved the inherited clustering effect associated with independent observations [18]. Furthermore, potential operator learning curve, mainly in the MM-group, could lead to operator-dependent bias which influences clinical outcomes. In this study, a single surgeon was utilized for all cases to standardize the operator’s dexterity.

This report is limited by a small enrollment sample and retrospective trial registration. Although the split-mouth design naturally yields greater statistical efficiency and higher relative effect sizes than independent parallel-group trials, interpreting the magnitude of these effects within a small cohort requires strict caution. Since effect size parameters can be artificially inflated in small sample sizes, our observed clinical differences must be interpreted as exploratory indices. In this report, significant differences in the primary outcome (ISQ) were detected despite a missing site and conservative power parameters, which suggests that the study was sufficiently powered to detect a substantial treatment effect. The short follow-up period in this report was adequate to observe the initial stages of osseointegration, yet it precludes long-term assessment of crestal bone maintenance and soft tissue stability. Consequently, CBCT-driven densitometry analysis carries its variation. Due to these constraints, larger and longer multi-center longitudinal trials remain essential to validate the generalizability of these clinical effects.

The overall compaction of the labial plate and preservation of the crestal alveolar process width achieved by both OD techniques could be beneficial in achieving better implant success in the maxillary anterior esthetic zone. The rotary-based OD offers supplementary benefits regarding the convenience of technique application, which may be particularly imperative in esthetically demanding regions, especially where thin cortical plates are present. Regarding the clinical relevance, the observed maintenance of implant stability ISQ values and preservation in labial plate thickness with the utilization of the rotary Densah burs may provide a safer biological buffer for immediate restoration in the esthetic zone, which is a growing patient demand. Similarly, the statistically significant difference in the labial plate thickness suggests its favorable influence on soft tissue stability, which points towards potentially more predictable esthetic outcome compared to the pulse technique.

## Conclusion

The osseodensification rotatory-based Densah Burs technique appeared to provide favorable clinical advantages over the pulse-based Magnetic Mallet in the maxillary anterior esthetic zone implant osteotomy preparation by yielding higher implant stability and labial plate thickness, suggesting potentially more predictable osteotomy compaction and autografting in this clinical context. However, a cautious interpretation of the overall intervention remains necessary, as it demonstrated statistically comparable outcomes regarding bone density and crestal width of the alveolar process. Furthermore, the occurrence of a labial plate fracture and subsequent implant failure within the MM-group underscores the critical importance of careful technique selection, though formal sensitivity modeling confirms that this single failure did not alter or skew the overarching statistical robustness of the primary stability findings.

## Supplementary Information

Supplementary Material 1: Figure S1. Descriptive grading of the Early Wound Healing (EWH) score and its contents. Figure S2. Clinical figure describing the osseodensification process with the utilization of rotary-based Densah Burs and pulse-based Magnetic Mallet. Figure S3. Sensitivity analysis outcome. Table S1: Baseline characteristics of the study participants. Table S2: Pain scores (VAS) values in the two Osseodensification groups. Table S3: Estimates of fixed effects for the various assessed parameters.

## Data Availability

All data generated or analyzed during this study are included in this published article.

## Abbreviations

OD: Osseodensification
DB: Densah bur
daN: Decanewton
SNOSE: Sequentially Numbered, Opaque, Sealed Envelopes
EWH: Early Wound Healing
CSR: Clinical signs of Re-epithelization
CSI: Clinical signs of Inflammation
⌀: Diameter
HU: Hounsfield Units
LMM: Linear Mixed-Effects Model
AIC: Akaike Information Criterion
RPM: Rotation Per Minute
MM: Magnetic Mallet
μs: Microsecond
CBCT: Cone-Beam Computed Tomography
VAS: Visual Analogue Scale
CSH: Clinical signs of Hemostasis
ISQ: Implant Stability Quotient
ROI: Region of Interest
GSV: Grey Scale Value
PDI: Prosthodontic Diagnostic Index
BIC: Bayesian Information Criterion
