# Supplementary material for: The effect of different osseodensification techniques on implant stability in the maxillary anterior esthetic zone. A split-mouth randomized clinical trial
Source: BMC Oral Health. 2026 Jun 5;26:1057. doi: 10.1186/s12903-026-08802-9 (PMC13270663; doi:10.1186/s12903-026-08802-9)
Supplement: Supplementary file 1 — Supplementary Material 1: Figure S1. Descriptive grading of the Early Wound Healing (EWH) score and its contents. Figure S2. Clinical figure describing the osseodensification process with the utilization of rotary-based Densah Burs and pulse-based Magnetic Mallet. Figure S3. Sensitivity analysis outcome. Table S1: Baseline characteristics of the study participants. Table S2: Pain scores (VAS) values in the two Osseodensification groups. Table S3: Estimates of fixed effects for the various assessed parameters. [file 12903_2026_8802_MOESM1_ESM.docx]

| **Supplementary Table 1: Baseline characteristics of the study participants.** | | | | | | | | | | |
| --- | --- | --- | --- | --- | --- | --- | --- | --- | --- | --- |
| **#** | **Site** | | **Allocation** | **Radiographic Analysis** | |  | **Implant Placement** | | |  |
|  |  |  |  | **Radiographic Available bone** | **Bone Density** |  | **Implant Size** | **Drilling Sequence** | **Procedural Complications** |  |
| **1** |  | | DB-Group | 5.8⌀ * 14 _L_ | D3 |  | 3.7⌀ * 10 _L_ | 2.0, 2.3, 2.5, 3.0, 3.3 | - |  |
|  | 1 |  |  |  |  |  |  |  |  |  |
|  |  |  |  |  |  |  |  |  |  |  |
|  |  | |  |  |  |  |  |  |  |  |
|  |  | | MM-Group | 5.5⌀ * 13 _L_ | D3 |  | 3.5⌀ * 10 _L_ | Pilot, 100P | Crack in the palatal side of the osteotomy during MM preparation |  |
|  |  | 1 |  |  |  |  |  |  |  |  |
|  |  |  |  |  |  |  |  |  |  |  |
|  |  | |  |  |  |  |  |  |  |  |
| **2** |  | | MM-Group | 4.8⌀ * 12 _L_ | D3 |  | INP | Pilot, 100P | Labial wall fracture occurred during MM preparation |  |
|  | 1 |  |  |  |  |  |  |  |  |  |
|  |  |  |  |  |  |  |  |  |  |  |
|  |  | |  |  |  |  |  |  |  |  |
|  |  | | DB-Group | 5.8⌀ * 13 _L_ | D3 |  | 3.7⌀ * 8 _L_ | 2.0, 2.3, 2.5, 3.0, 3.3 | - |  |
|  | 2 |  |  |  |  |  |  |  |  |  |
|  |  |  |  |  |  |  |  |  |  |  |
|  |  | |  |  |  |  |  |  |  |  |
| **3** |  | | MM-Group | 5.9⌀ * 14 _L_ | D2 |  | 3.5⌀ * 8 _L_ | Pilot, 100P | - |  |
|  | 1 |  |  |  |  |  |  |  |  |  |
|  |  |  |  |  |  |  |  |  |  |  |
|  |  | |  |  |  |  |  |  |  |  |
|  |  | | DB-Group | 6.1⌀ * 13 _L_ | D3 |  | 3.7⌀ * 8 _L_ | 2.0, 2.3, 2.5, 3.0, 3.3 | - |  |
|  |  | 1 |  |  |  |  |  |  |  |  |
|  |  |  |  |  |  |  |  |  |  |  |
|  |  | |  |  |  |  |  |  |  |  |
| **4** |  | | DB-Group | 5.8⌀ * 12 _L_ | D2 |  | 3.7⌀ * 10 _L_ | 2.0, 2.3, 2.5, 3.0, 3.3 | - |  |
|  |  | 1 |  |  |  |  |  |  |  |  |
|  |  |  |  |  |  |  |  |  |  |  |
|  |  | |  |  |  |  |  |  |  |  |
|  |  | | MM-Group | 6.0⌀ * 14 _L_ | D2 |  | 3.7⌀ * 10 _L_ | Pilot, 100P, 160P | - |  |
|  | 3 |  |  |  |  |  |  |  |  |  |
|  |  |  |  |  |  |  |  |  |  |  |
|  |  | |  |  |  |  |  |  |  |  |
| **5** |  | | MM-Group | 5.0⌀ * 13 _L_ | D3 |  | 3.5⌀ * 10 _L_ | Pilot, 100P | - |  |
|  | 2 |  |  |  |  |  |  |  |  |  |
|  |  |  |  |  |  |  |  |  |  |  |
|  |  | |  |  |  |  |  |  |  |  |
|  |  | | DB-Group | 5.9⌀ * 13 _L_ | D3 |  | 3.7⌀ * 10 _L_ | 2.0, 2.3, 2.5, 3.0, 3.3 | - |  |
|  | 1 |  |  |  |  |  |  |  |  |  |
|  |  |  |  |  |  |  |  |  |  |  |
|  |  | |  |  |  |  |  |  |  |  |
| **6** |  | | DB-Group | 6.2⌀ * 12 _L_ | D2 |  | 4.0⌀ * 10 _L_ | 2.0, 2.3, 2.5, 3.0, 3.3, 3.5 | - |  |
|  | 1 |  |  |  |  |  |  |  |  |  |
|  |  |  |  |  |  |  |  |  |  |  |
|  |  | |  |  |  |  |  |  |  |  |
|  |  | | MM-Group | 6.1⌀ * 13 _L_ | D2 |  | 3.5⌀ * 10 _L_ | Pilot, 100P | - |  |
|  |  | 1 |  |  |  |  |  |  |  |  |
|  |  |  |  |  |  |  |  |  |  |  |
|  |  | |  |  |  |  |  |  |  |  |
| **7** |  | | DB-Group | 5.9⌀ * 13 _L_ | D3 |  | 3.7⌀ * 10 _L_ | 2.0, 2.3, 2.5, 3.0, 3.3 | - |  |
|  |  | 2 |  |  |  |  |  |  |  |  |
|  |  |  |  |  |  |  |  |  |  |  |
|  |  | |  |  |  |  |  |  |  |  |
|  | ` | | MM-Group | 5.8⌀ * 14 _L_ | D3 |  | 3.7⌀ * 10 _L_ | Pilot, 100P, 160P | - |  |
|  | 1 |  |  |  |  |  |  |  |  |  |
|  |  |  |  |  |  |  |  |  |  |  |
|  |  | |  |  |  |  |  |  |  |  |
| DB: Densah Bur, MM: Magnetic mallet, ⌀: Diameter, L: Length, INP: Implant Not Placed, | | | | | | | | | | |

| **Supplementary Table 2: Pain scores (VAS) values in the two Osseodensification groups.** | | | | | |
| --- | --- | --- | --- | --- | --- |
| **VAS** | | **DB (n=7)** | **MM (n=6)** | ***P*** | |
| **7^th^ day** | Mean ±SD | 7.00 ±0.82 | 7.67 ±1.75 | 0.373 | |
| **10^th^ day** |  | 4.29 ±1.11 | 6.17 ±1.17 | **0.016*** | |
| **14^st^ day** |  | 1.29 ±1.11 | 2.67 ±1.86 | 0.071 | |
| ***P*** | | **<0.001*** | **<0.001*** |  | |
| *Statistically significant difference at p value<0.05. | | | | | |

| **Supplementary Table 3: estimates of fixed effects for the various assessed parameters.** | | | | | | | | | | |
| --- | --- | --- | --- | --- | --- | --- | --- | --- | --- | --- |
| **Parameters** | **Estimate** | | **Standard Error** | **df** | **t value** | ***P* value** | | **95% CI** | | |
|  |  |  |  |  |  |  |  | **LB** | **UB** | |
| **Implant stability (ISQ)** | | | | | | | | | | |
| Intercept | 59.84 | | 3.87 | 17.47 | 15.46 | **<0.001^*^** | | 51.69 | 67.99 | |
| Groups _(DB vs MM)_ | 14.87 | | 4.25 | 16.81 | 3.50 | **0.003^*^** | | 5.90 | 23.85 | |
| Time. _(primary ISQ vs secondary ISQ)_ | -10.83 | | 4.36 | 16.39 | -2.48 | **0.024^*^** | | -20.07 | -1.60 | |
| Groups _(_**_DB_**_)_ x Time _(primary ISQ)._ | 6.26 | | 5.95 | 16.39 | 1.05 | 0.308 | | -6.32 | 18.84 | |
| **Bone Density (Hu)** | | | | | | | | | | |
| Intercept | 693.65 | 48.81 | | 18.82 | 14.21 | **<0.001^*^** | 591.43 | | | 795.87 |
| Groups _(DB vs MM)_ | 55.35 | 56.57 | | 16.61 | .98 | 0.342 | -64.22 | | | 174.92 |
| Time. _(Immediate Postoperative vs 6-months’ Postoperative)._ | 135.50 | 58.19 | | 16.12 | 2.33 | **0.033^*^** | 12.22 | | | 258.78 |
| Groups _(DB)_ x Time _(Immediate Postoperative)._ | 17.36 | 79.30 | | 16.12 | 0.22 | 0.829 | -150.64 | | | 185.35 |
| **Crestal width of the alveolar process (mm)** | | | | | | | | | | |
| Intercept | 5.41 | | 0.17 | 21.97 | 31.67 | **<0.001^*^** | | 5.06 | 5.77 | |
| Groups _(DB vs MM)_ | -0.11 | | 0.23 | 16.93 | -0.50 | 0.625 | | -0.60 | 0.37 | |
| Time. _(Immediate Postoperative vs 6-months’ Postoperative)._ | 0.53 | | 0.24 | 16.29 | 2.24 | **0.040^*^** | | 0.03 | 1.04 | |
| Groups _(DB)_ x Time _(Immediate Postoperative)._ | -0.42 | | 0.33 | 16.29 | -1.28 | 0.219 | | -1.10 | 0.27 | |
| **Overall labial bone thickness (mm)** | | | | | | | | | | |
| Intercept | 0.98 | | 0.16 | 20.05 | 6.32 | **<0.001^*^** | | 0.66 | 1.31 | |
| Groups _(DB vs MM)_ | 0.70 | | 0.19 | 16.97 | 3.71 | **0.002^*^** | | 0.30 | 1.09 | |
| Time. _(Immediate Postoperative vs 6-months’ Postoperative)._ | 0.19 | | 0.19 | 16.46 | .97 | 0.345 | | -0.22 | 0.60 | |
| Groups _(DB)_ x Time _(Immediate Postoperative)._ | -0.02 | | 0.26 | 16.46 | -.08 | 0.937 | | -0.58 | 0.54 | |
| *Statistically significant difference at P <0.05, df: degree of freedom | | | | | | | | | | |

| 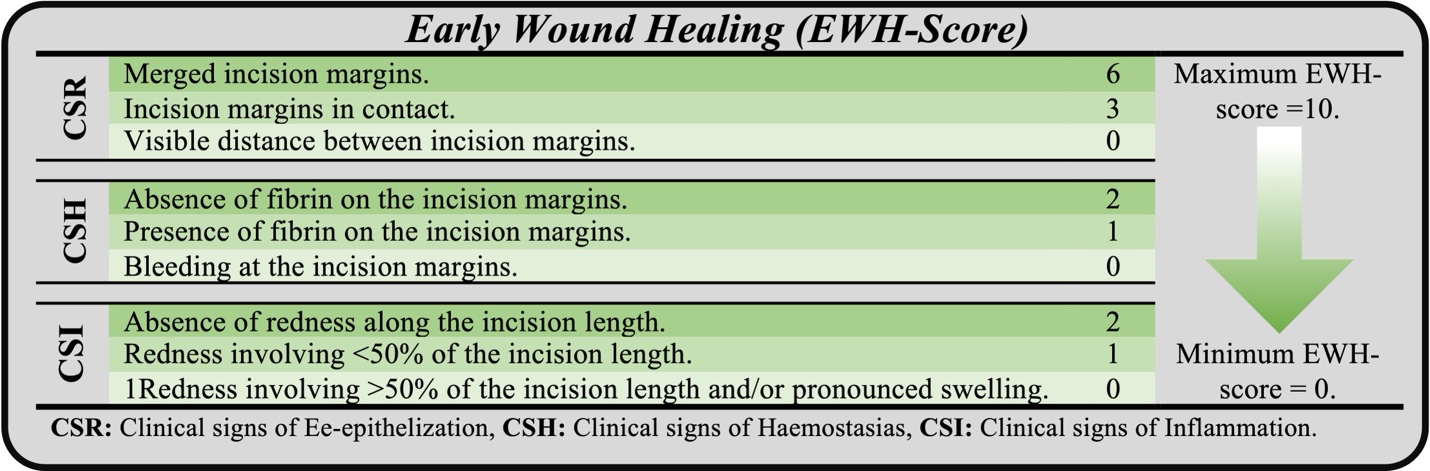 |
| --- |
| **Supplementary Figure 1.** Descriptive grading of the Early Wound Healing (EWH) score and its contents |

| 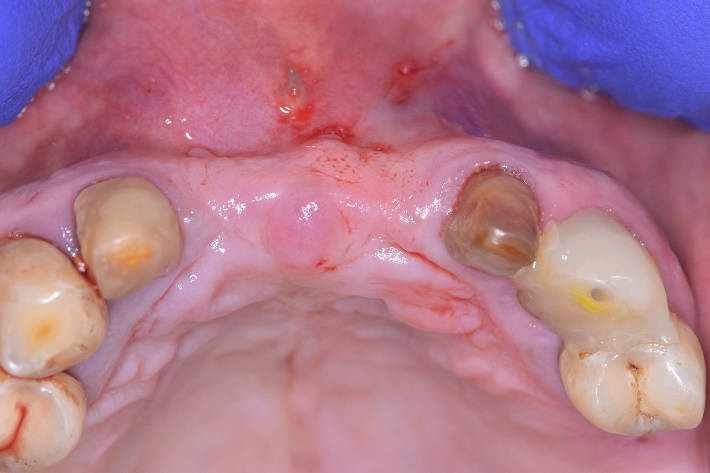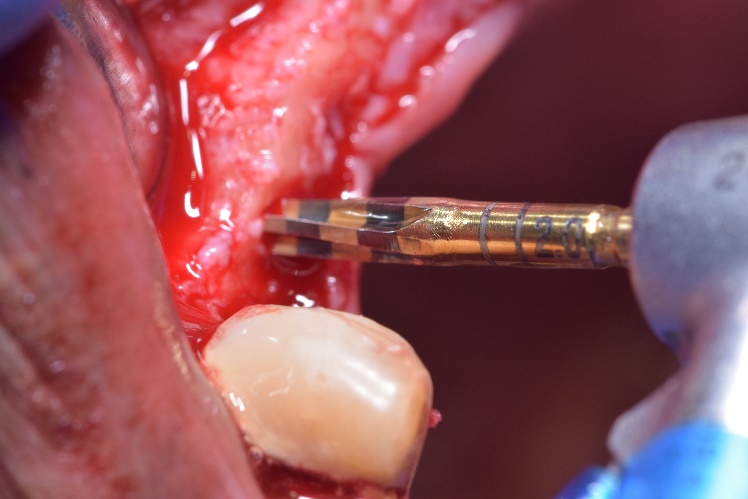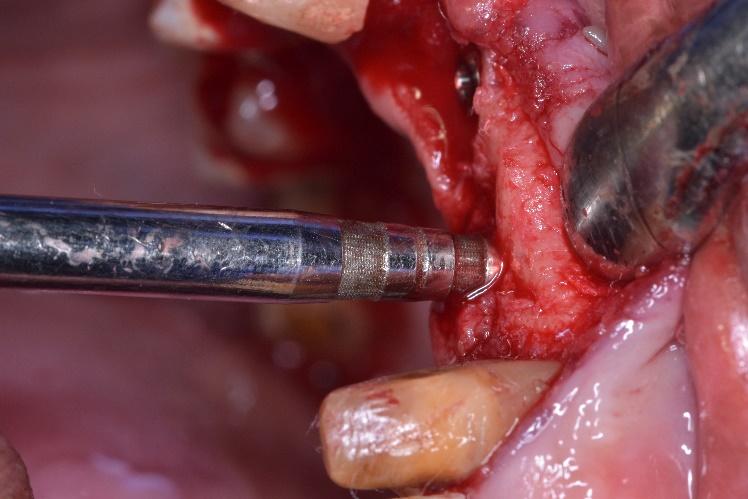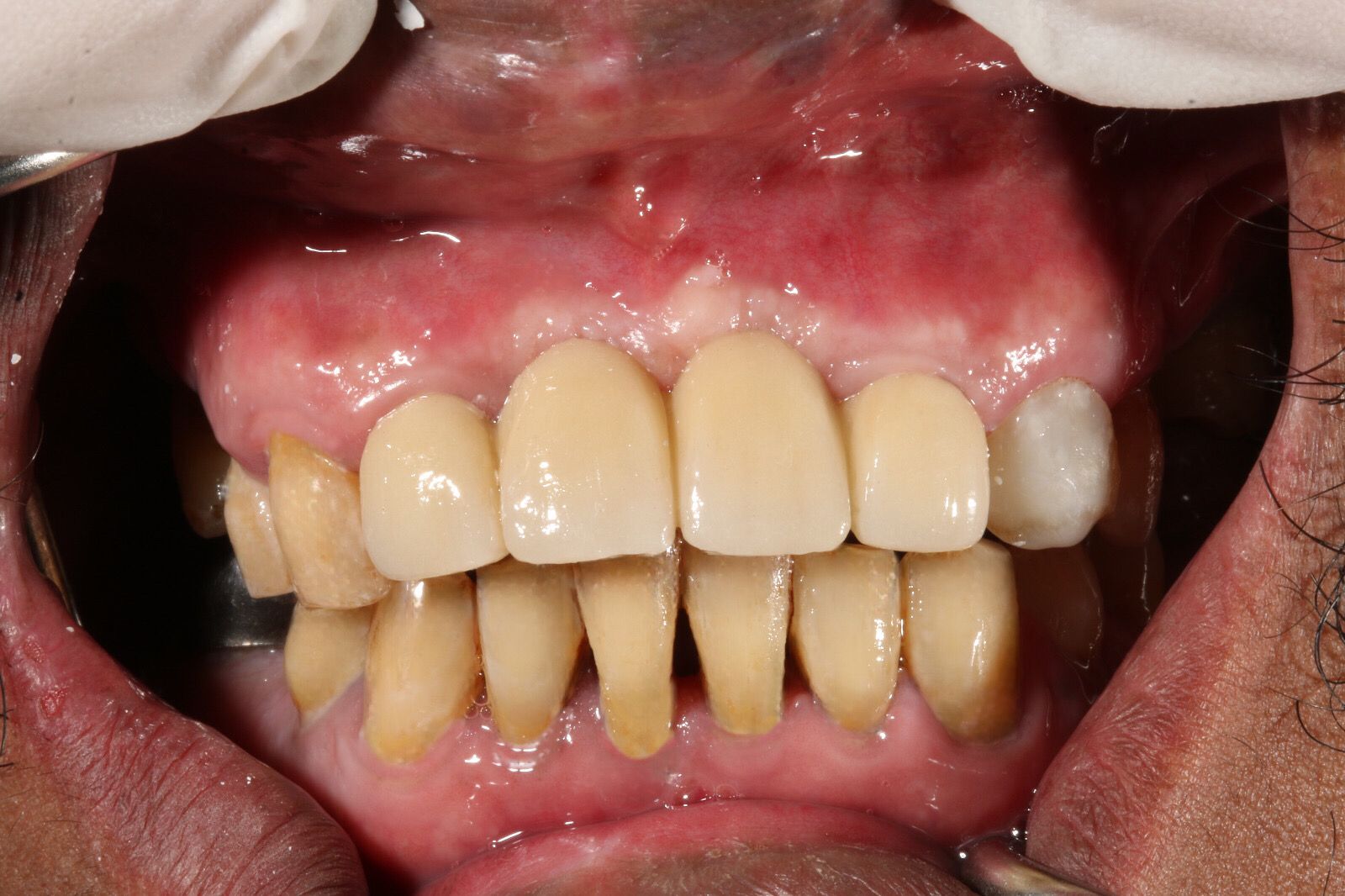 |
| --- |
| **Supplementary Figure 2.** Clinical figure describing the osseodensification process with the utilization of rotary-based Densah Burs and pulse-based Magnetic Mallet. |

***Sensitivity analysis outcome***

A formal paired sensitivity analysis for the primary outcome (Implant Stability) to evaluate the impact of the single failed case. The sensitivity analysis was conducted twice.

- The first model was conducted through a complete-case framework, omitting the patient with the failed implant:

The DB-group demonstrated significantly higher ISQ scores compared to the MM-group [Excluding the Failed Case; (75.17±8.70 vs. 59.83±4.31; ***P*= 0.009**)].

- The second model was conducted through the framework that the patient with the failed implant was retained within the pairwise analysis to maintain the complete split-mouth dataset.

The DB-group demonstrated significantly higher ISQ scores compared to the MM-group [Including the Failed Case; (74.71±7.97 vs. 57.00±8.16; ***P*=0.0033**)].

The consistency in both models with the LMM, the outcome of the sensitivity analysis confirms that our conclusions are methodologically robust and not skewed by the handling of the failed case.

| 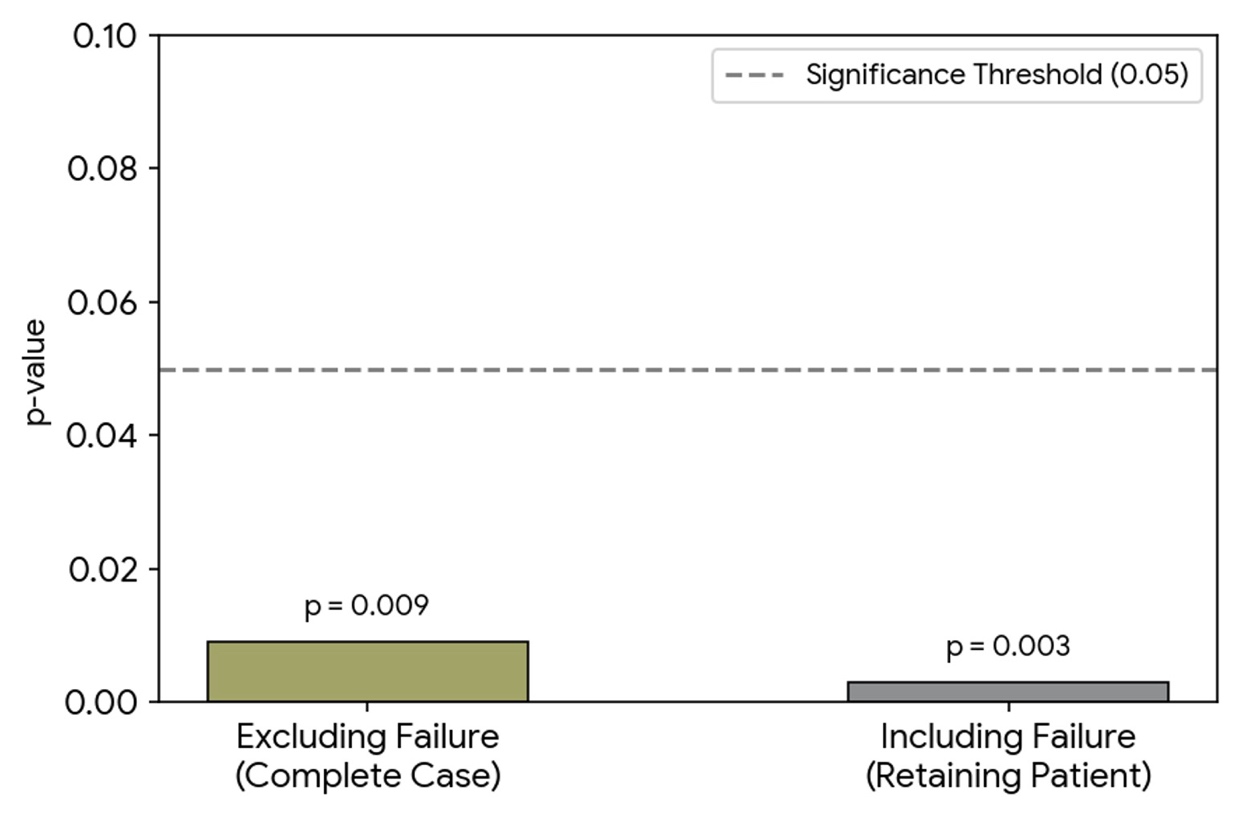 |
| --- |
| **Supplementary Figure 3.** Sensitivity analysis outcome |
